# Revealing the key modules and potential prognostic markers of gastric cancer transformation based on weighted gene co-expression networks

**DOI:** 10.3389/fgene.2025.1613682

**Published:** 2025-11-25

**Authors:** Heng Li, Wen Li, Zhen Yang, Haiyu Liu, Xiaoping Zhang, Yufeng Zhao, Hao Gu

**Affiliations:** 1 Institute of Basic Research in Clinical Medicine, China Academy of Chinese Medical Sciences, Beijing, China; 2 School of Pharmacy, Lanzhou University, Lanzhou, China; 3 Tianjin University of Traditional Chinese Medicine, Tianjin, China; 4 Data Center of Traditional Chinese Medicine, China Academy of Chinese Medical Sciences, Beijing, China

**Keywords:** WGCNA, key gene, key module, precancerous lesions of the stomach, gastric cancer

## Abstract

**Background:**

This study aims to identify key modules and targets during the transition from gastric precancerous lesions to gastric cancer by performing weighted gene co-expression network analysis (WGCNA) on gene microarray datasets from the Gene Expression Omnibus (GEO) database containing gastritis, gastric cancer and precancerous lesions, providing insights for early intervention in gastric cancer.

**Methods:**

Transcriptomic data from precancerous lesions (including low-grade and high-grade intraepithelial neoplasia) and early gastric cancer were analyzed using differential gene analysis, WGCNA, and survival analysis. Critical modules and genes associated with disease progression were identified. The prognostic value and expression changes of these genes were evaluated, and their expression patterns across disease states were validated in external datasets to confirm key genes involved in the inflammation-cancer transformation into gastric cancer.

**Results:**

WGCNA identified four key modules: pink, purple, red, and magenta. The first three modules were most strongly associated with low-grade intraepithelial neoplasia, high-grade intraepithelial neoplasia, and early gastric cancer, respectively, while magenta was linked to all three stages. Functional analysis reveals: Pink module: Enriched in inflammation-related pathways. Purple module: Involved in chemical carcinogenesis and beta-alanine metabolism. Red module: Associated with immune response and inflammation, participating in NF-kappa B and Toll-like receptor signaling pathways. Magenta module: Linked to complement activation and immune response, enriched in cytokine-cytokine receptor interaction and chemokine signaling pathways. Core genes are filtered based on gene significance (GS > 0.2) and module membership (MM > 0.8). Among 20 shared core genes across disease stages, 13 genes (e.g., *FCRL3*,*EFEMP1*,*ANKRD29*,*STOX2*) were identified as unfavorable prognostic factors for gastric cancer. External validation confirmed consistent expression patterns of these genes in training and validation datasets, with all four genes (*FCRL3*, *EFEMP1*, *ANKRD29*, *STOX2*) significantly correlating with poor prognosis.

**Conclusion:**

WGCNA reveals modules associated with gastric precancerous lesions and cancer progression. *FCRL3*, *EFEMP1*, *ANKRD29*, and *STOX2* may serve as potential biomarkers for monitoring the transition from precancerous lesions to gastric cancer, offering insights into the mechanisms of gastric carcinogenesis and supporting early diagnosis and intervention strategies.

## 1 Introduction

According to the 2024 National Cancer Report released by the National Cancer Center, gastric cancer (GC) ranks third in cancer-related mortality (Zheng et al., 2024), posing a significant threat to public health as a major chronic non-communicable disease. Approximately 95% of gastric cancers are adenocarcinomas, with intestinal-type gastric cancer (IGC) and diffuse-type gastric cancer (DGC) representing the two major histological subtypes. These subtypes exhibit significant differences at the molecular level. Notably, IGC exhibits a higher tumor mutational burden and stronger association with genomic instability compared to DGC, underscoring its relevance for studying inflammation-driven carcinogenesis. Gastric carcinogenesis typically follows a multistep progression from chronic gastritis (CG) to precancerous lesions—such as atrophic gastritis (AG), intestinal metaplasia (IM), and intraepithelial neoplasia (IN)—and ultimately to gastric cancer (GC), a process well described by the Correa cascade (Correa and Piazuelo, 2012). Accumulating evidence indicates that this progression is most prominent in IGC, which accounts for the majority of GC cases and is closely associated with *Helicobacter* pylori-induced chronic inflammation (Wang et al., 2022). IN, a critical precancerous stage, is histologically classified into low-grade (LGIN) and high-grade (HGIN) based on the degree of cellular atypia, with HGIN carrying a significantly increased risk of malignant transformation.

Early intervention in the precancerous stages is crucial to decelerate disease progression and prevent malignant transformation (Bray et al., 2018; Zhou et al., 2019; Yan-Rui et al., 2024). The concept of “inflammation-cancer transformation” refers to the pathological continuum whereby persistent inflammation promotes tumor initiation and progression through a series of coordinated molecular and cellular events.

During this transformation process, chronic inflammatory stimulation prompts the secretion of various cytokines (such as IL-6), which drives the polarization of macrophages from the M1 type to the M2 type (Wei et al., 2024). This polarization, together with the activation of cancer-associated fibroblasts (CAFs) (Cheng et al., 2023), jointly forms a tumor-promoting microenvironment. This immune microenvironment provides favorable conditions for the immune escape of tumor cells and supports the clonal expansion of transformed epithelial cells, thereby forming a self-reinforcing cycle that promotes malignant transformation.

**FIGURE 1:**
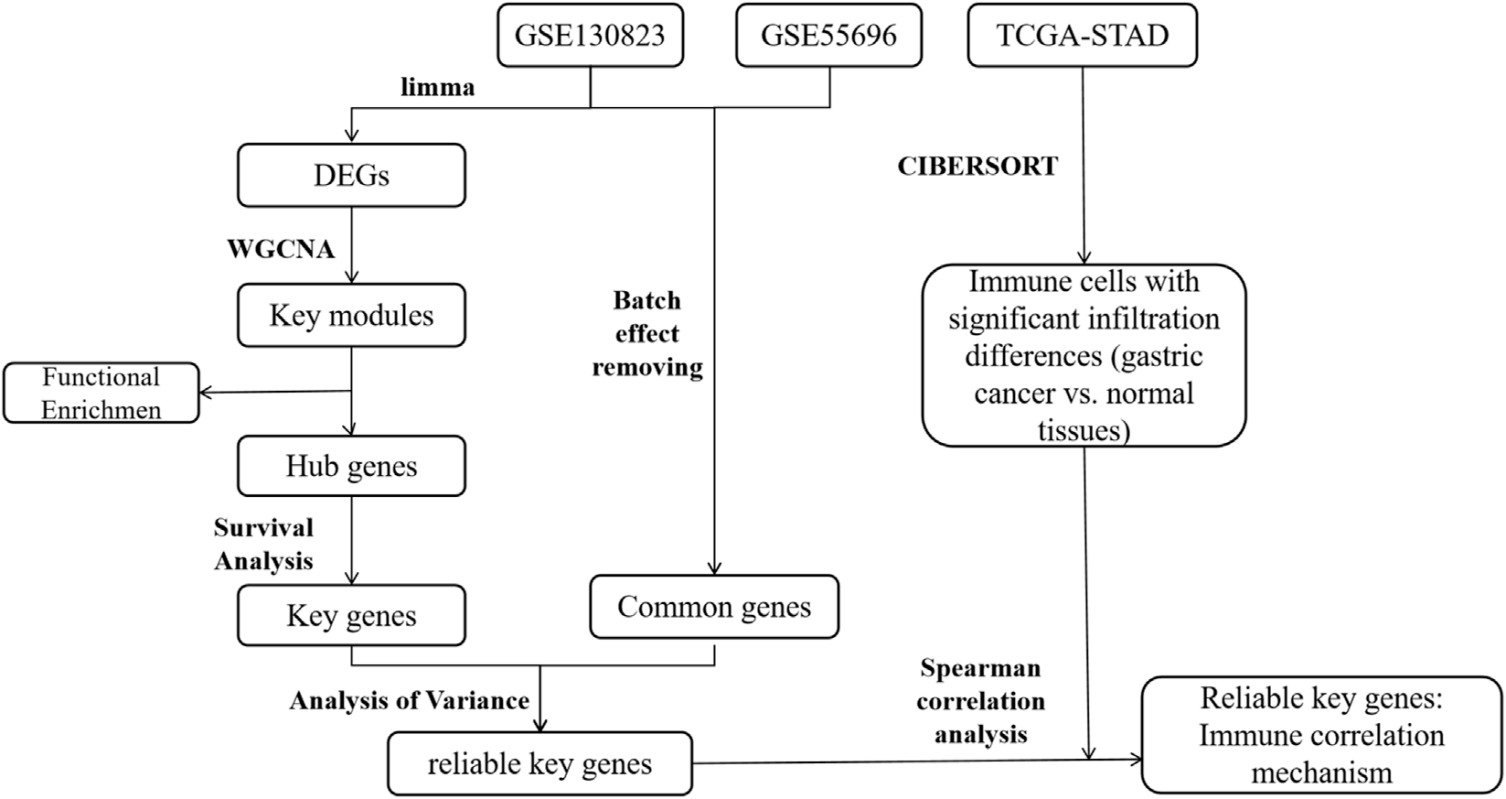
Flow chart of the analysis process conducted in this study.

Although WGCNA, a systems biology approach effective in identifying functionally coordinated gene modules, has been applied in gastric cancer research (Wu et al., 2025; Zhang et al., 2025), these studies have primarily focused on tumor tissues alone, with limited integration of precancerous stages. Consequently, the dynamic gene co-expression networks driving the stepwise progression from chronic gastritis to cancer remain poorly characterized. In this study, we integrated multi-stage transcriptomic datasets from patients with intestinal-type gastric cancer-related lesions, including CG, low-grade intraepithelial neoplasia (LGIN), high-grade intraepithelial neoplasia (HGIN), and early gastric cancer (EGC). Using WGCNA, we systematically characterized the dynamic gene expression patterns underlying inflammation-cancer transformation. We further performed KEGG pathway enrichment, survival analysis, and external validation to identify key genes and pathways. Importantly, we coupled this with comprehensive immune cell infiltration analysis to explore how the remodeling of the tumor immune microenvironment regulates this transition. The overall study design is illustrated in Figure 1.

## 2 Materials and methods

### 2.1 Dataset acquisition

We obtain microarray datasets (GSE130823 and GSE55696) (Xu et al., 2014; Zhang et al., 2020) from Gene Expression Omnibus (GEO) database. GSE130823 serves as the training set, and GSE55696 serves as the validation set. Detailed information about these datasets is provided in Table 1. FPKM (Fragments per kilobase of transcript per million mapped reads)-formatted gene expression data and corresponding clinical data for the STAD project are obtained from the TCGA database by the Sangerbox 3.0 (Chen et al., 2024) platform for subsequent immune cell infiltration analysis and clinical feature correlation analysis.

**TABLE 1:** Datasets information.

GEO ID	Platform	Samples	Group
GSE130823	GPL17077	CG:LGIN:HGIN:EGC = 47:17:14:16	Experience
GSE55696	GPL6480	CG:LGIN:HGIN:EGC = 19:19:20:19	Validation

### 2.2 Dataset preprocessing

The raw gene expression matrix undergoes preprocessing, including filtering of low-expression genes (retaining only genes with expression values > 1 in at least 10% of samples) and averaging expression levels of duplicated gene entries to generate a standardized gene expression matrix. To integrate two datasets and mitigate batch effects, the ComBat function from the R package “sva” is applied for batch effect correction. To evaluate the effectiveness of correction, box plots and principal component analysis (PCA) plots are generated for the data before and after batch adjustment.

### 2.3 Identification of differentially expressed genes (DEGs)

Using the limma tool in the Sangerbox 3.0 platform, we perform differential analysis on the training set with |log2FC| > 0.58 and p < 0.05 as the criteria for screening differentially expressed genes. We obtain DEGs for LGIN, HGIN, and EGC using CG as the control group and visualize the results. Additionally, we import the DEGs from the three disease states into the Bioinformatics platform for further visualization of their distribution.

### 2.4 Weighted co-expression network analysis (WGCNA) and key module selection

WGCNA was performed to identify co-expression modules associated with the inflammation-to-cancer transition in gastric carcinogenesis. The analysis was conducted on DEGs identified across the disease continuum (CG → LGIN → HGIN → EGC) from the training dataset, to enrich for genes with potential functional relevance.

Gene expression data were imported into Sangerbox 3.0, and an unsigned co-expression network was constructed. Genes with a mean standard deviation proportion >50% were retained, and outlier samples were removed prior to network construction. A soft-thresholding power (β) was selected to approximate a scale-free topology (*R* ^2^ > 0.85). The topological overlap matrix (TOM), which measures network interconnectedness by integrating both direct and indirect gene-gene correlations, was calculated. Hierarchical clustering and dynamic tree-cutting (minimum module size = 30, merge cut height = 0.25, sensitivity = 3) were used to define gene modules.

For each module, the module eigengene (ME; the first principal component of the standardized gene expression matrix) was computed to represent its overall expression pattern. The Pearson correlation between ME and each clinical trait (disease stage) was calculated to assess module–phenotype associations. Modules showing strong positive or negative correlations (|r| > 0.2, p < 0.05) with specific stages (LGIN, HGIN, EGC) or across multiple stages were selected as key modules for downstream functional and hub gene analysis.

### 2.5 Hub gene selection

Gene Significance (GS) measures gene–phenotype correlation; higher GS indicates stronger biological relevance. Module Membership (MM) is defined by the Pearson correlation coefficient between a gene’s expression profile and the ME. This metric quantifies the strength of gene–module association; |MM| near one signifies high connectivity within the module. Using established thresholds (|MM| > 0.8 and |GS| > 0.2) (Tang et al., 2018b), we identify hub genes in phenotype-associated modules. Genes differentially expressed across all three disease states are prioritized as key drivers of gastric carcinogenesis.

### 2.6 Functional enrichment analysis

GO and KEGG enrichment analyses of key modules are performed on the Sangerbox 3.0 platform. The platform uses gene GO annotations from the R package org. Hs.e.g.,.db (version 3.1.0) as background, maps genes to this set, and obtains the latest KEGG Pathway gene annotations as another background for mapping. Enrichment analysis is carried out using the R package clusterProfiler (version 3.14.3) to get gene set enrichment results. The minimum gene set is set to 5 and the maximum to 5,000, with p < 0.05 indicating statistical significance.

### 2.7 Survival analysis

To evaluate the prognostic value of key genes in gastric cancer, we first obtain TCGA-STAD (Stomach Adenocarcinoma) survival information data using the gene names from the Sangerbox 3.0 platform. We then perform survival analysis using the “survival” package in R and visualize the results with the “survminer” package. Gene expression data are log-transformed [log (x + 0.001)], and samples with zero expression or survival time less than 30 days are excluded. The optimal expression cutoff for each gene is determined using the maxstat package (0.7.26), limiting group sizes to between 25% and 75% of the total cohort. Patients are stratified into high-expression and low-expression groups, and Kaplan-Meier curves are generated using the survfit function of the survival package. Survival differences are assessed by the log-rank test, with statistical significance set at p < 0.05.

### 2.8 External validation

To assess the reliability of the key genes, we extract their expression profiles from both the training and validation datasets. Prior to comparative analysis, the ComBat method is applied to correct for batch effects and ensure data comparability. We then perform Analysis of Variance on each dataset separately, with the aim of examining whether the expression trends of the key genes across different disease states are consistent between the validation and training sets.

### 2.9 Correlation analysis of key genes with immune cell infiltration and clinical characteristics

The functional status of immune cells in the tumor microenvironment (TME) is critically important for tumor progression and patient prognosis. To investigate the association between key genes and immune cell infiltration, this study utilizes RNA-seq data from the TCGA-STAD cohort. Prior to immune infiltration analysis, the dataset was filtered to retain only intestinal-type EGC samples. This filtering step was performed to ensure consistency with our previous analyses, which were conducted exclusively on this subset of samples, thereby guaranteeing the reliability and comparability of the immune infiltration results. Immune cell infiltration was quantified using the CIBERSORT algorithm, and samples with a P-value <0.05 were selected as a reliable dataset for downstream analysis. To identify immune cells with significant infiltration differences between gastric cancer tissues and adjacent normal tissues, comparative statistical analysis was performed. To explore the potential mechanisms through which key genes regulate immune infiltration in gastric cancer, Spearman correlation analysis was applied to evaluate the relationship between the expression levels of key genes and the infiltration proportions of 22 immune cell types. Furthermore, to elucidate the association between key genes and clinical characteristics, statistical analysis was performed to examine the expression differences of the key gene across different tumor grades.

### 2.10 Statistical methods

Data are analyzed using the SPSSPRO online platform (Scientific Platform Serving for Statistics, 2021) and visualized with GraphPad Prism 8.0.2. For key genes, we first test normality and homogeneity of variance. Genes with normal distribution are analyzed using one-way ANOVA, while those with normality but not homogeneity of variance are analyzed using Welch’s ANOVA. LSD is used for multiple comparisons between groups, with p < 0.05 considered statistically significant.

## 3 Results

### 3.1 Differential expression gene analysis results

**FIGURE 2:**
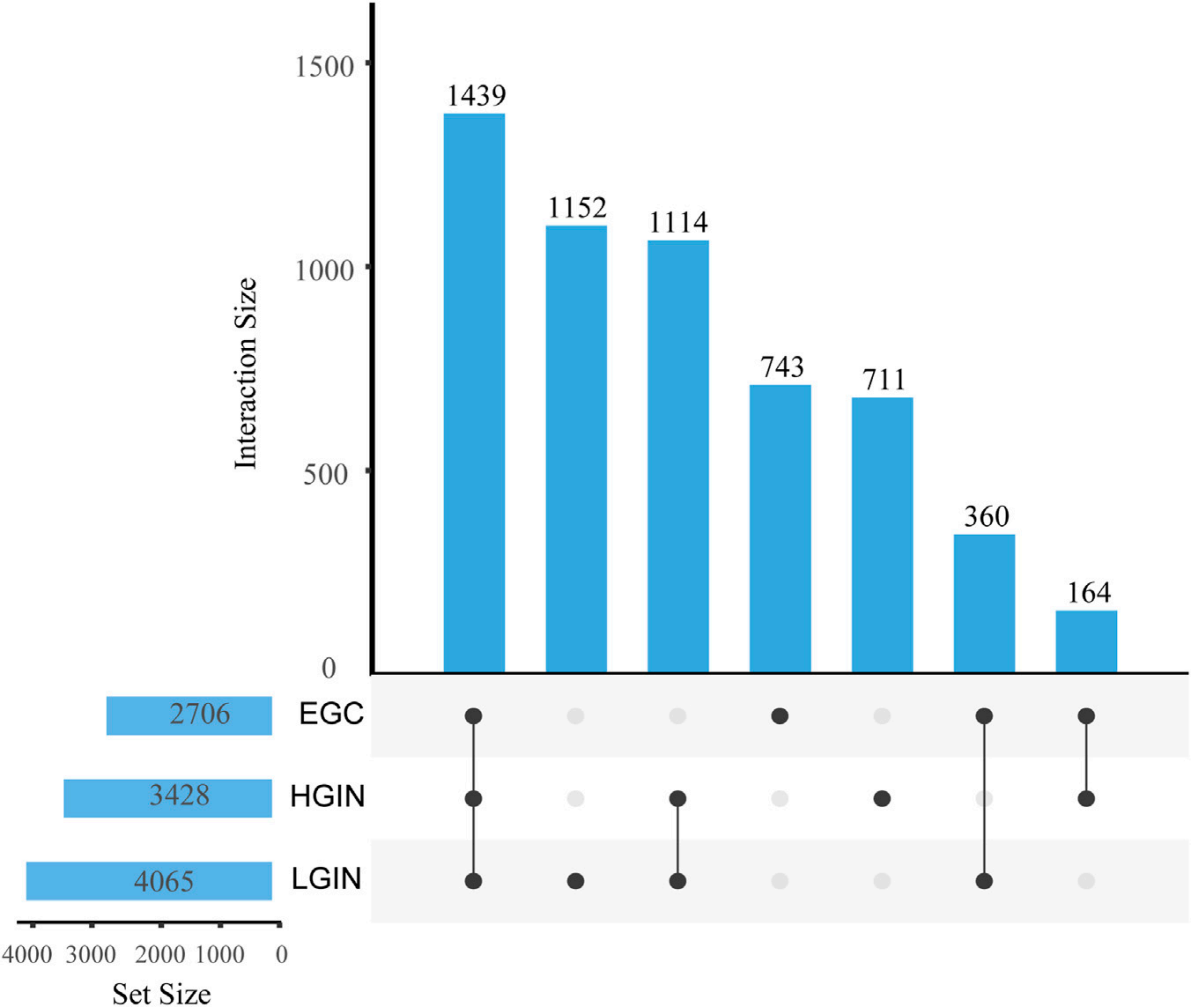
Intersection and specific DEGs in EGC, HGIN, and LGIN compared to CG. The bar height represents the number of genes in each intersection set, the numbers above the x-axis indicate the total number of differentially expressed genes for each disease group, and the numeric labels on the connecting lines denote the number of genes in the corresponding overlapping or unique category.

**FIGURE 3:**
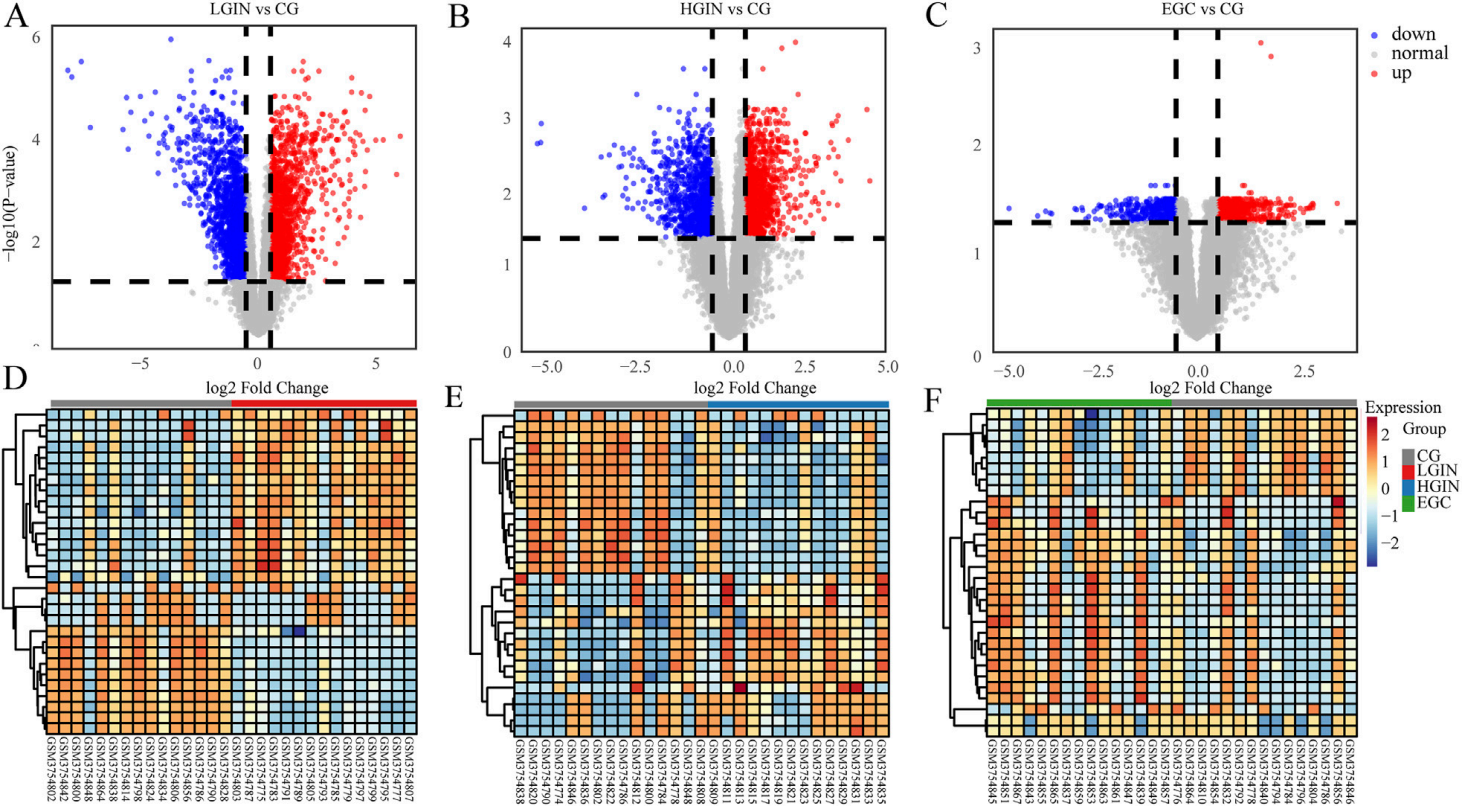
Volcano plot and heatmap analysis. **(A–C)** Volcano plots showing the DEGs of LGIN, HGIN, and EGC compared with CG. **(D–F)** Heatmaps of the differentially expressed genes under the corresponding conditions. In **(A–C)**, blue dots represent downregulated DEGs, red dots represent upregulated DEGs, and gray dots represent non-differentially expressed genes. In the heatmaps **(D–F)**, the color gradient from blue to red indicates the gene expression level from low to high.

Compared with the CG group, we identify 4,065 DEGs in LGIN (2030 downregulated, 2035 upregulated), 3,428 DEGs in HGIN (1,618 downregulated, 1810 upregulated), and 2,706 DEGs in EGC (1,075 downregulated, 1,631 upregulated), as shown in the volcano plots (Figure 2). The UpSet plot (Figure 3) reveals 1,439 common DEGs across the three disease states after intersection. This observation can be attributed to more extensive mucosal remodeling and activation of molecular pathways during the precancerous stages, whereas intestinal-type gastric cancer, upon acquisition of specific driver mutations or signaling pathways, undergoes clonal selection that leads to a reduction in the number of differentially expressed genes.

### 3.2 WGCNA results and key module selection

**FIGURE 4:**
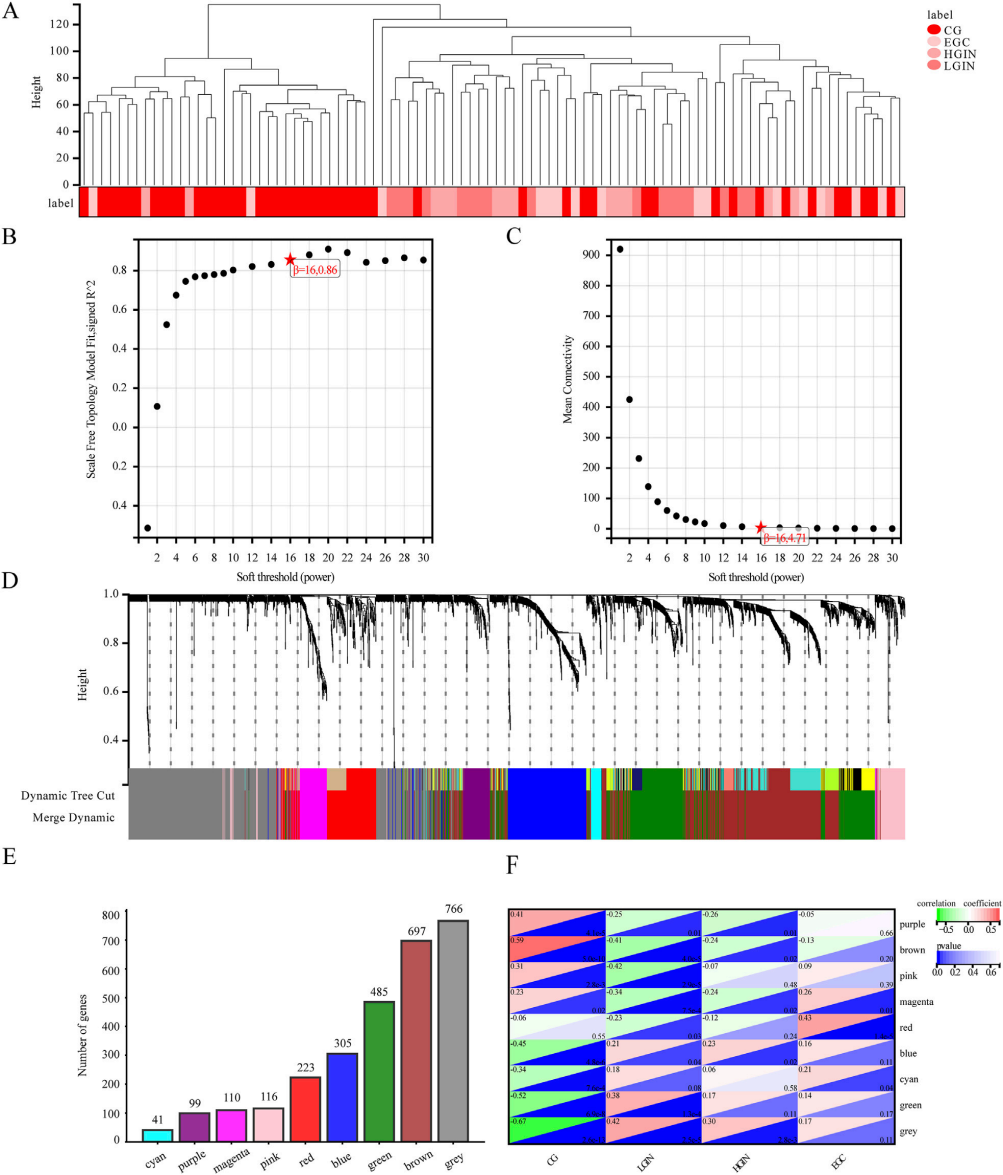
Analysis results of the weighted co-expression network module. **(A)** Sample clustering dendrogram. **(B)** Soft threshold determination. **(C)** Average connectivity. **(D)** Dynamic tree cutting to classify and merge modules with high similarity to gene clustering trees. **(E)** Number of genes in different modules. **(F)** Heatmap of module-phenotype correlation. Each square in the heatmap consists of two triangles. The upper-left triangle represents the correlation between different disease states and the module, with red indicating a positive correlation and green indicating a negative correlation. The lower-right triangle represents the p-value of the correlation, where darker blue indicates a smaller p-value.

With a correlation coefficient threshold of 0.85, the soft threshold β is determined to be 16 (Figures 4A-C). At this parameter setting, the modules divided under such conditions are more consistent with the characteristics of a scale-free network and thus possess greater biological significance. Setting the minimum module size at 30 and sensitivity at 3, we identify 9 modules (Figure 4D): cyan, purple, magenta, pink, red, blue, green, brown, and grey. These modules contain 41, 99, 110, 116, 223, 305, 485, 697, and 766 genes, respectively. The grey module comprises unassigned genes, while the other 8 modules contain the genes as shown in Figure 4E.

The module–phenotype correlation analysis (Figure 4F) indicates unique correlations of several modules with EGC development. The modules most associated with LGIN, HGIN, and EGC are the pink (r = −0.42, p < 0.05), purple (r = −0.26, p < 0.05), and red (r = 0.43, p < 0.05), respectively. The magenta module is correlated with all three disease states (r = −0.34 to 0.26, p < 0.05). A positive correlation indicates that the overall expression profile of the module increases with disease progression (e.g., higher in EGC than in CG), suggesting potential oncogenic or progression-promoting roles. In contrast, a negative correlation implies that the module’s expression decreases during progression (e.g., lower in advanced stages), which may reflect loss of gastric differentiation or tumor-suppressive functions. Thus, we select the pink, purple, red, and magenta modules as key modules for subsequent analysis.

There are 45 hub genes in the pink module for LGIN, 35 in the purple module for HGIN, 85 in the red module for EGC, and 76, 49, and 59 hub genes for LGIN, HGIN, and EGC, respectively, in the magenta module.

### 3.3 Functional enrichment of key modules

**FIGURE 5:**
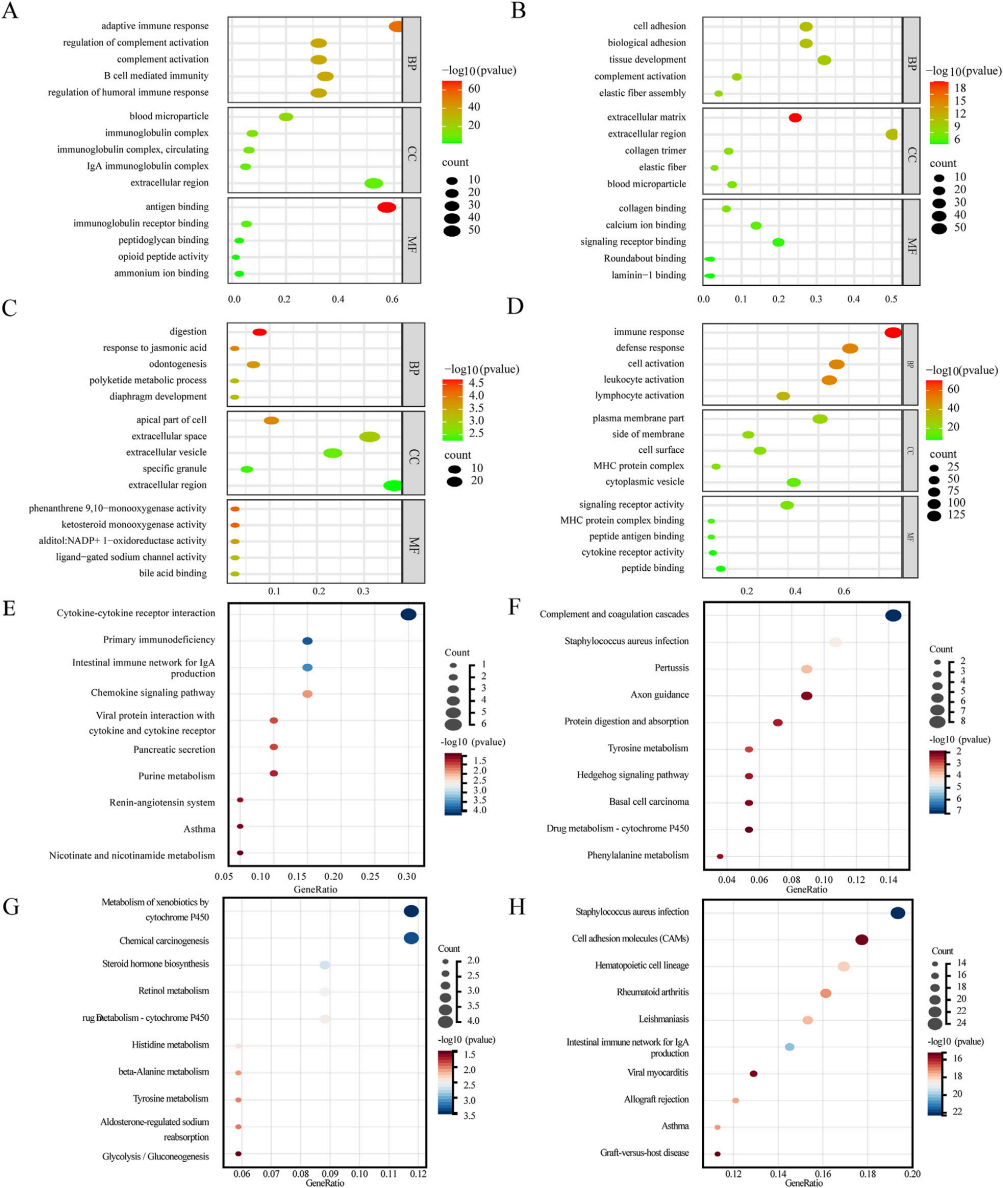
Results of key module enrichment analysis. GO enrichment results for the **(A)** magenta, **(B)** pink, **(C)** purple, and **(D)** red modules. KEGG pathway enrichment results for the **(E)** magenta, **(F)** pink, **(G)** purple, and **(H)** red modules. Bubble size represents the number of genes within each module enriched for a given term or pathway. Color intensity reflects the level of enrichment significance, expressed as −log_10_ (p-value), with darker red indicating higher statistical significance (lower p-value) and greener shades indicating lower significance (higher p-value).

The pink module (linked to LGIN) is associated with cell adhesion, angiogenesis, and inflammation, and is involved in pathways like basal cell carcinoma and proteoglycans in cancer (Figures 5B,F). The purple module (linked to HGIN) is related to doxorubicin metabolism, TNF secretion regulation, and cell response to reactive oxygen species, and is involved in pathways such as chemical carcinogenesis and drug metabolism (Figures 5C,G). The red module (linked to EGC) is associated with immune response and inflammation, and is involved in pathways like NF-kappa B signaling and Toll-like receptor signaling (Figures 5D–H). The magenta module (linked to all three disease states) is related to complement activation and immune response, and is involved in pathways such as cytokine-cytokine receptor interaction and chemokine signaling (Figures 5A,E).

### 3.4 Key genes and survival analysis

**FIGURE 6:**
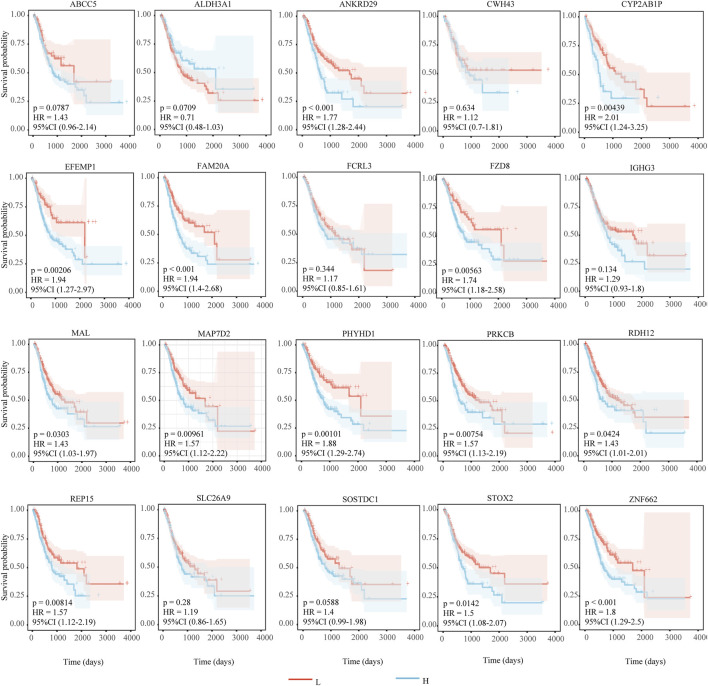
Results of survival analysis of key genes. The horizontal axis represents survival time (days), and the vertical axis represents survival probability. Red indicates low expression of the gene, and blue indicates high expression of the gene. HR > 1 indicates that high expression of the gene is unfavorable for the prognosis of gastric cancer patients. P < 0.05 and HR > 1 indicate that the adverse effect of the gene on the prognosis of gastric cancer patients is statistically significant.

We identify 20 key genes by intersecting hub genes from WGCNA modules with differentially expressed genes common to all three disease states (Table 2). Survival analysis reveals that 13 of these genes are significantly associated with poor prognosis in gastric cancer patients (log-rank test, p < 0.05, HR > 1; Figure 6), and are thus highlighted as prognostic candidates (Table 3).

**TABLE 2:** Key module genes.

Key module	Key gene
Pink	*EFEMP1*
Purple	*ABCC5, FZD8, ALDH3A1, MAL, SLC26A9, REP15, ANKRD29, RDH12, PHYHD1, SOSTDC1, STOX2, MAP7D2, FAM20A, ZNF662, CWH43, CYP2AB1P*
Red	*FCRL3, PRKCB*
Magenta	*IGHG3*

**TABLE 3:** The results of survival analysis.

Key gene	HR	95% CI	P Value
*ANKRD29*	1.77	1.28–2.44	0.0006
*CYP2AB1P*	2.01	1.24–3.25	0.0044
*EFEMP1*	1.94	1.27–2.97	0.0021
*FAM20A*	1.94	1.40–2.68	0.0001
*FZD8*	1.74	1.18–2.58	0.0056
*MAL*	1.43	1.03–1.97	0.0303
*MAP7D2*	1.57	1.12–2.22	0.0096
*PHYHD1*	1.88	1.29–2.74	0.0010
*PRKCB*	1.57	1.13–2.19	0.0075
*RDH12*	1.43	1.01–2.01	0.0424
*REP15*	1.57	1.12–2.19	0.0081
*STOX2*	1.50	1.08–2.07	0.0142
*ZNF662*	1.80	1.29–2.50	0.0005

### 3.5 Validation results of key genes

**FIGURE 7:**
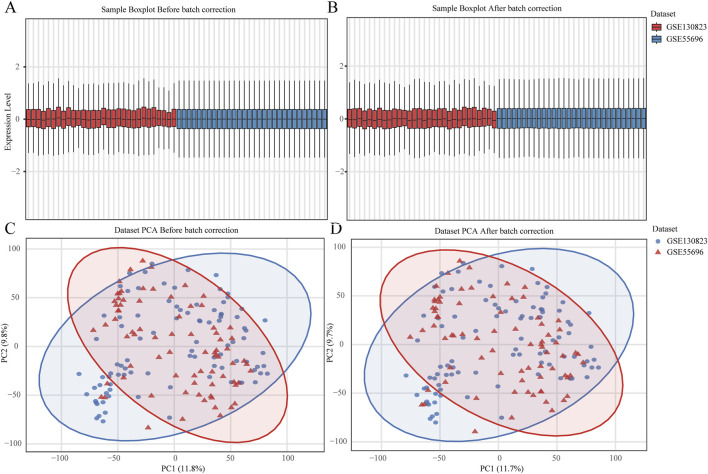
Batch Effect Evaluation of GSE130823 and GSE55696 Datasets. **(A,B)** Box plots of gene expression values before (left) and after (right) batch effect correction. **(C,D)** Principal component analysis (PCA) plots before (left) and after (right) batch effect correction.

**FIGURE 8:**
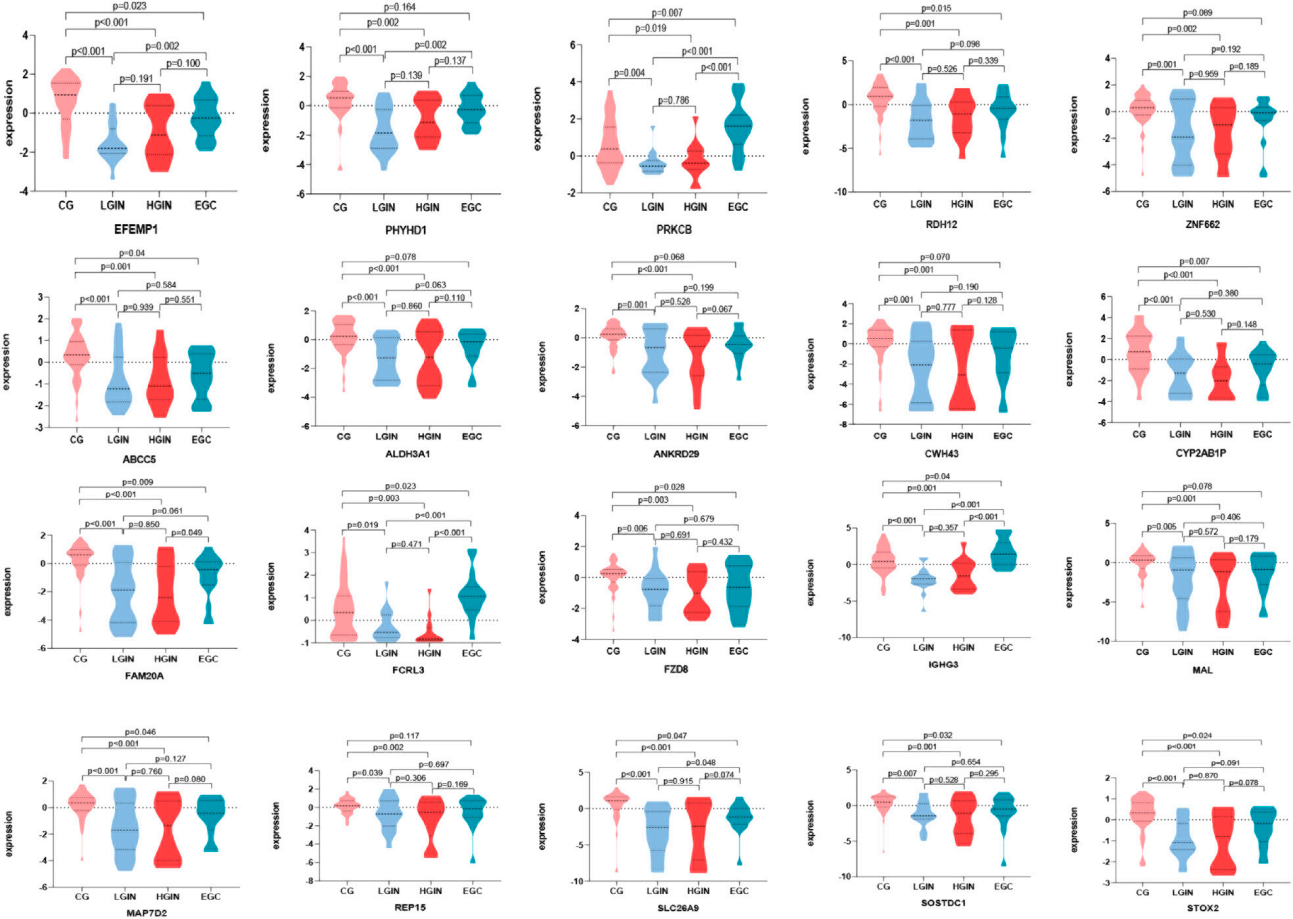
Expression of key genes in the GSE130823 dataset. Different colors on the horizontal axis represent different disease stages, and the vertical axis represents gene expression levels. Each graph shows the expression levels of key genes across different disease stages, where P < 0.05 indicates that the difference in expression between two disease stages is statistically significant.

**FIGURE 9:**
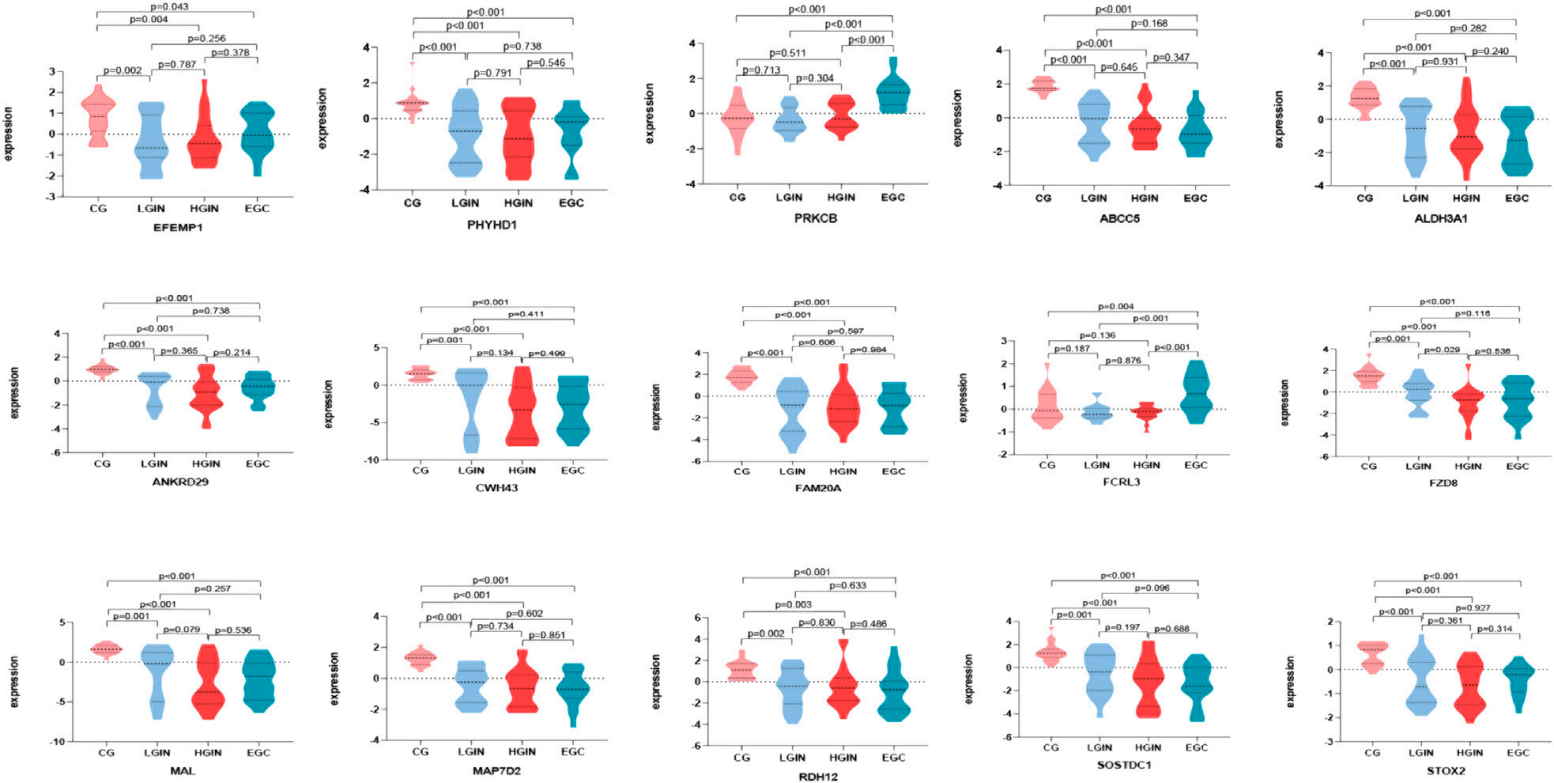
Expression of key genes in GSE55696 dataset. Different colors on the horizontal axis represent different disease stages, and the vertical axis represents gene expression levels. Each graph shows the expression levels of key genes across different disease stages, where P < 0.05 indicates that the difference in expression between two disease stages is statistically significant.

Batch effect correction and visualization are performed on the two datasets, and it is found that there is almost no batch effect between them (Figure 7). When analyzing the key genes in the validation set, 5 key genes are not present. After conducting analysis of variance on the remaining 15 key genes, it is observed that the expression levels of these 15 key genes show significant differences across different disease stages (p < 0.05). According to the analysis results, in the training set, *IGHG3*, *FCRL3*, and *PRKCB* have the highest expression in EGC, while other genes have the highest expression in CG (Figure 8). Five key genes *(IGHG3*, *CYP2AB1P*, *REP15*, *SLC26A9*, *ZNF662*) are not annotated or expressed in the validation set. For the remaining 15 genes, expression analysis shows that *FCRL3* and *PRKCB* have the highest expression in EGC, while the other genes have the highest expression in CG (Figure 9).

### 3.6 Association of key genes with immune infiltration and clinical features

**FIGURE 10:**
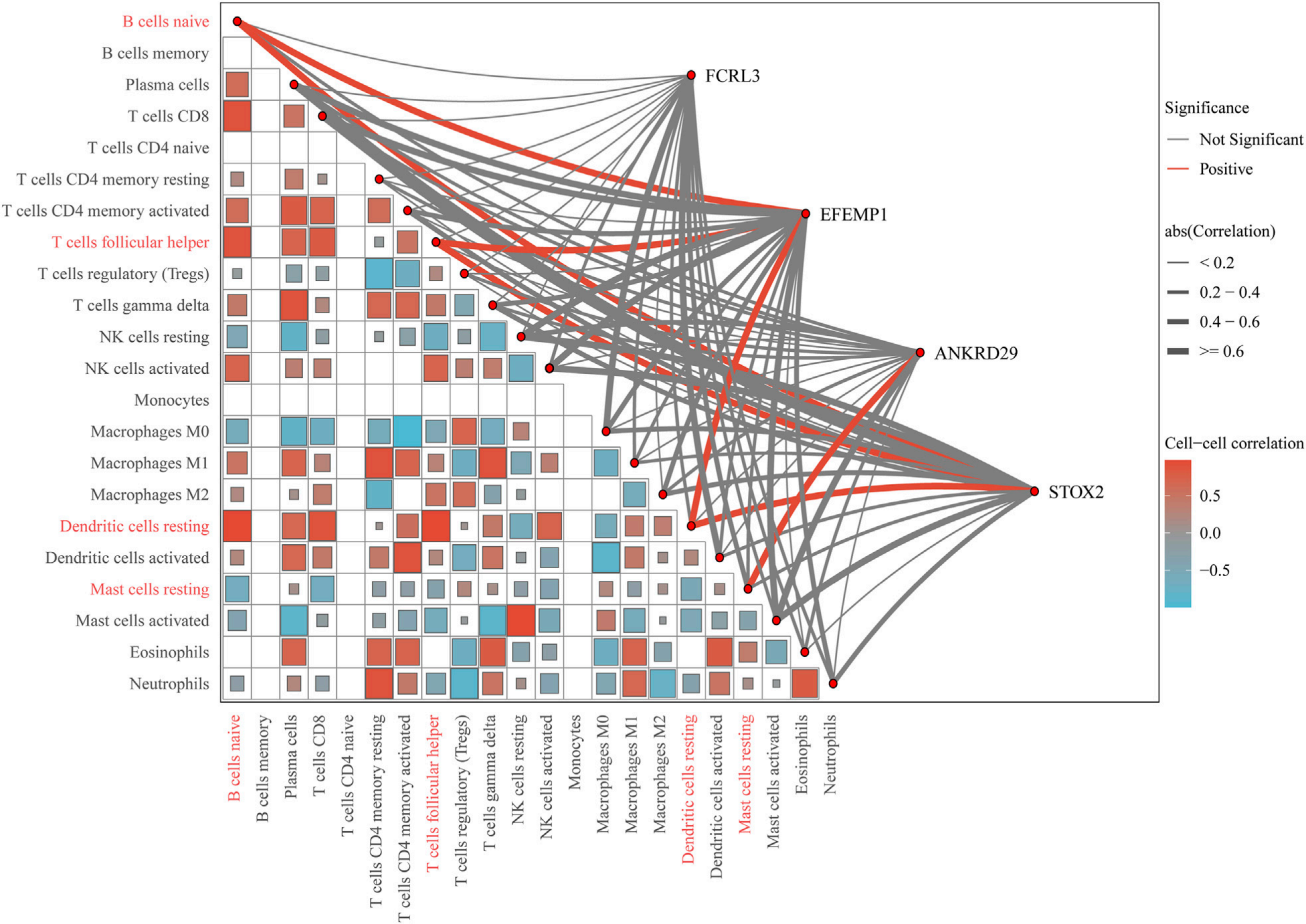
Comprehensive correlation network of key genes and immune cell infiltration. The outer chord diagram visualizes the correlations between the key genes (*FCRL3, EFEMP1, ANKRD29, STOX2*) and 22 immune cell types. Red chords represent significant positive correlations, blue chords represent significant negative correlations, and gray chords represent non-significant associations. The thickness of each chord is proportional to the absolute value of the correlation coefficient, with thicker chords indicating stronger relationships. The inner triangular heatmap illustrates the pairwise correlation matrix among the immune cell types themselves. The color intensity and scale bar (ranging from −0.5 to 0.5) represent the strength and direction of these cell-cell correlations.

CIBERSORT analysis of the TCGA-STAD dataset reveals distinct immune infiltration patterns in gastric cancer. Compared to normal tissues, tumor tissues exhibit elevated infiltration of resting dendritic cells, while showing reduced levels of B cells naive, T cells follicular helper, and mast cells resting. Spearman correlation analysis demonstrates that the key gene *EFEMP1* is positively correlated with B cells naive and dendritic cells resting; *ANKRD29* is positively correlated with mast cells resting; and *STOX2* shows positive correlations with dendritic cells resting, B cells naive, and T cells follicular helper (Figure 10).

**FIGURE 11:**
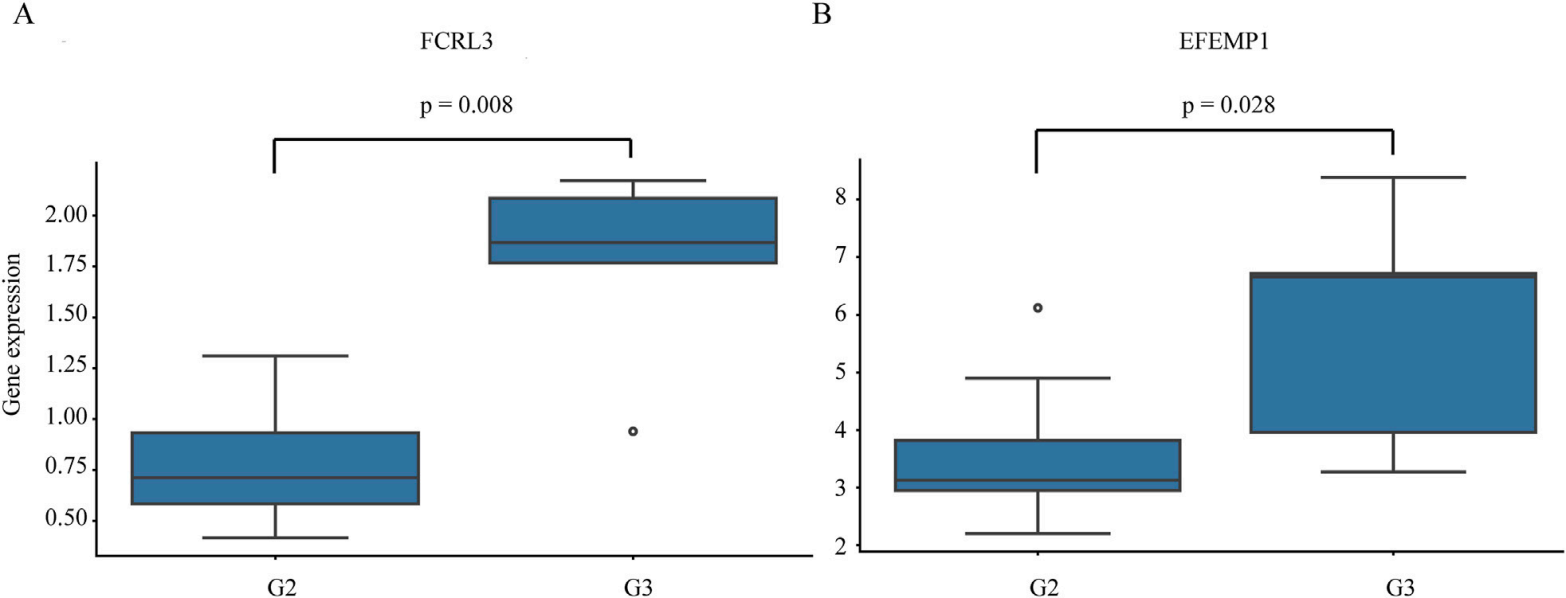
Expression of key genes across different gastric cancer grades. Horizontal axis: Tumor grades (G2, G3; G2 = moderately differentiated, intermediate malignancy; G3 = poorly differentiated, high malignancy with significant cellular atypia). Vertical axis: Key gene expression levels. **(A)** FCRL3; **(B)** EFEMP1. P < 0.05 indicates statistically significant differences in gene expression among grades.

To evaluate clinical relevance, we analyze the association between key gene expression and tumor grade. Notably, the expression levels of *FCRL3* and *EFEMP1* are significantly correlated with tumor differentiation, with both genes showing significantly lower expression in G2 (moderately differentiated) tumors compared to G3 (poorly differentiated) tumors (Figure 11). No significant differences are observed for other key genes across tumor grades. These findings suggest that high expression of *FCRL3* and *EFEMP1* may be associated with tumor dedifferentiation and disease progression, highlighting their potential as prognostic biomarkers in gastric cancer.

## 4 Discussion

This study aims to explore key molecular mechanisms in the progression of gastritis to gastric cancer and identify potential early - diagnosis biomarkers. We first use the limma package to screen DEGs in LGIN, HGIN, and EGC. However, since disease development usually involves multiple genes rather than single one (Barabási et al., 2011; Hanahan and Weinberg, 2011), we further perform WGCNA to capture key modules associated with these disease stages, which allows systematic analysis of gene co-regulation, suitable for studying complex disease mechanisms (Langfelder and Horvath, 2008; Tang et al., 2018a).

Through WGCNA, we identify key modules and genes related to the three disease stages in gastric carcinogenesis. These key genes, with high connectivity in modules, may play a crucial role in disease development (Zhang and Horvath, 2005).

Notably, the magenta module shows a U-shaped correlation pattern across gastric carcinogenesis: positively correlated with chronic gastritis (CG; r = 0.23, p = 0.02) and early gastric cancer (EGC; r = 0.26, p = 0.01), but negatively correlated with low-grade (r = −0.34, p = 7.5 × 10^−5^) and high-grade intraepithelial neoplasia (HGIN; r = −0.24, p = 0.02). This dynamic trajectory suggests stage-specific roles of B cell–mediated immunity.

The module is enriched in B cell activation, plasma cell differentiation, and humoral immunity pathways, including intestinal immune network for IgA production and primary immunodeficiency. This highlights the central involvement of B-lineage cells.

In CG, the module likely reflects protective immune responses against pathogens like *H. pylori*. Its downregulation in LGIN/HGIN may indicate impaired B-cell function or immune evasion during dysplasia. In contrast, reactivation in EGC could drive a pro-tumorigenic microenvironment through tertiary lymphoid structure formation, immunoglobulin-mediated complement activation, and recruitment of suppressive myeloid cells.

Thus, the magenta module represents a context-dependent immune axis—shifting from protective immunity to tumor-promoting inflammation—highlighting the dual roles of adaptive immunity in gastric cancer development.

To evaluate the clinical significance of key genes, survival analysis is conducted. Among the 20 key genes, 13 are significantly associated with poor patient prognosis, indicating they might be risk factors. Suppressing overexpression could be beneficial for gastric cancer patients.

Univariate analysis of the 20 key genes, combined with their expression in the validation set, revealed that four genes (e.g., *FCRL3, EFEMP1, ANKRD29, STOX2*) showed expression trends highly consistent with the original dataset. Among these, survival analysis for *FCRL3* (HR = 1.17, 95% CI 0.85–1.61, p = 0.343) shows no statistically significant difference. While *EFEMP1* (HR = 1.94, 95% CI 1.27–2.97, p < 0.001), *ANKRD29* (HR = 1.77, 95% CI 1.28–2.44, p < 0.001), and *STOX2* (HR = 1.50, 95% CI 1.08–2.07, p = 0.014) are all associated with a positive prognostic value for gastric cancer patients. Eleven genes (e.g., *ABCC5, ALDH3A1, SOSTDC1,* etc.) show inconsistent expression trends compared to the original dataset, possibly due to sample heterogeneity.

The protein encoded by the *FCRL3* gene is primarily expressed in B lymphocytes and enhances the activation of both NF-κB and MAPK signaling pathways in TLR9-stimulated B cells (Li et al., 2013). The MAPK pathway consists of multiple core kinases and is divided into several cascades, including ERK and JNK(Lei et al., 2022; Yuan et al., 2024). Through the ERK/MEK/RAF cascade, it promotes migration and invasion of gastric cancer cells, while also facilitating cell proliferation and differentiation—processes that may contribute to gastritis–cancer transformation. The JNK pathway, on the other hand, is associated with cellular stress response and apoptosis, and may promote tumor cell survival and proliferation in gastric cancer through activation of transcription factors such as c-Jun. NF-κB represents a class of transcription factors whose aberrant activation has been linked to increased invasiveness, metastasis, and chemotherapy resistance in gastric cancer (Lei et al., 2022).

*EFEMP1*, a fibulin family member containing epidermal growth factor, functions in the extracellular matrix. It has diverse roles in various cancers, acting as both an oncogene and a tumor suppressor (Yang et al., 2013). In lung cancer cells, downregulated *EFEMP1* is linked to tumor growth and invasion (Lang et al., 2015). It also inhibits hepatocellular carcinoma cell migration (Dou et al., 2016), with its downregulation being associated with increased migration and related to *ERK1/2*, *MMP2*, and *MMP9* expression. Given its potential to inhibit tumor growth and invasion in different cancers, *EFEMP1* - targeted therapies may have clinical potential in future gastric cancer treatment.

*ANKRD29* belongs to the ankyrin repeat domain (*ANKRD*) protein family, which is widely involved in protein - protein interactions and signal transduction in eukaryotes. It may influence patients’ treatment responses by modulating the immune microenvironment and drug sensitivity, suggesting potential applications in immunotherapy and chemotherapy (Zhao et al., 2023a). Specifically, *ANKRD29* acts as a tumor - suppressor gene, particularly in non - small cell lung cancer (NSCLC) tumorigenesis. It inhibits NSCLC progression by regulating cell proliferation, migration, and apoptosis. Reduced *ANKRD29* expression significantly enhances NSCLC cell proliferation and migration, while restoring its expression suppresses tumor growth by inhibiting the cell cycle and modulating relevant signaling pathways (Zhao et al., 2023a).

*STOX2* encodes a transcription factor. When inhibited by miR - 30a, it activates multiple tumor - progression - related signaling pathways, including *ERK*, *AKT*, and *P38*. Activation of these pathways promotes cell survival, proliferation, and metastasis (Guo et al., 2022).

To further explore the potential roles of these four key genes in the transition from gastritis to gastric cancer, we performed an in-depth interpretation of the above findings. CIBERSORT analysis based on the TCGA-STAD dataset revealed that resting dendritic cells were significantly more enriched in early gastric cancer tissues compared to normal gastric mucosa, suggesting their involvement in the early remodeling of the tumor immune microenvironment. Previous studies have reported elevated levels of resting dendritic cells in high-risk molecular subtypes of gastric cancer and their association with poor prognosis (Zhao et al., 2023b). Although dendritic cells can contribute to antigen presentation within tertiary lymphoid structures (TLSs) and initiate anti-tumor immunity, their resting or immature state is often linked to impaired T-cell activation and the induction of immune tolerance (Bai et al., 2022). Thus, the accumulation of resting dendritic cells may undermine immune surveillance and facilitate tumor progression.

Spearman correlation analysis shows that the expression of *EFEMP1* and STOX2 was positively correlated with the infiltration level of resting dendritic cells, implying that these genes may contribute to the recruitment or maintenance of an immunosuppressive microenvironment. Furthermore, clinical stratification analysis reveals that *EFEMP1* expression is significantly higher in G3 (poorly differentiated) tumors than in G2 (moderately differentiated) tumors, reinforcing its association with tumor dedifferentiation and aggressive phenotypes.

Collectively, *EFEMP1* may play a pro-tumorigenic role in gastric carcinogenesis by promoting the accumulation of resting dendritic cells, potentially serving as a molecular link between chronic inflammation and malignant transformation, as well as a candidate prognostic biomarker.

## 5 Conclusion

This study integrates multi-stage transcriptomic data and employs WGCNA to systematically identify potential key genes driving the inflammation-to-cancer transformation from a dynamic perspective of “gastritis–precancerous lesion–early gastric cancer” progression. We not only identify 20 key genes consistently differentially expressed throughout this transformation process but also validate four highly robust core genes—*FCRL3*, *EFEMP1*, *ANKRD29*, and *STOX2*—with significant prognostic impact using external datasets. The expression patterns of these genes show high consistency across multiple independent cohorts, and their elevated expression is significantly correlated with poorer overall survival in gastric cancer patients, highlighting their substantial as novel prognostic biomarkers and targets for clinical translation.

In the NF-κB/MAPK signaling axis, *ANKRD29* directly regulates the MAPK pathway (Zhao et al., 2023a), while *FCRL3* and *EFEMP1* exert their effects primarily through indirect mechanisms. The immunosuppressive functions of *FCRL3* (e.g., restricting T cell activation) may create an inflammatory environment in the tumor microenvironment (TME), thereby indirectly promoting NF-κB-mediated inflammatory signaling. *EFEMP1* potentially affects the MAPK pathway (e.g., ERK signaling) via the EGFR axis (Ying et al., 2021; Shen et al., 2023); however, its secretory property may regulate the extracellular matrix of the TME and interfere with NF-κB-controlled stromal cell interactions (Almeida et al., 2023). As a MAPK regulator, the low expression of *ANKRD29* may amplify pro-tumor signals in the TME of non-small cell lung cancer (NSCLC).

Within the TME, NF-κB governs the crosstalk between immunity, inflammation, and tumor progression (Cao et al., 2024; Alipourgivi and Lu, 2025). Through immunomodulation (e.g., suppressing T cell function), *FCRL3* may reinforce the immunosuppressive phenotype of the TME and enhance NF-κB-dependent drug resistance (Li et al., 2024). The secretion of EFEMP1 promotes metastasis and TME remodeling, which may synergize with NF-κB-driven TME changes (Almeida et al., 2023). In contrast, high expression of *ANKRD29* inhibits the proliferation and migration of NSCLC cells, improves anti-tumor immunity by enhancing T cell cytotoxicity (Zhao et al., 2022a), and may counteract the pro-tumor effects of the NF-κB/MAPK axis.

*FCRL3* influences the TME indirectly mainly through immunomodulation, which may be associated with the NF-κB inflammatory axis; *EFEMP1* promotes tumor metastasis and TME remodeling via EGFR-mediated signaling; *ANKRD29* directly regulates the MAPK pathway, exerts a tumor-suppressive effect in NSCLC, and enhances immune responses in the TME. Within the NF-κB/MAPK signaling axis, the roles of these three molecules contribute to cancer development and TME dynamics primarily through independent or indirect pathways. Furthermore, integrating immune infiltration analysis and key gene-clinical correlation results, we propose that in gastric cancer, *FCRL3* may partially suppress tumor invasion and metastasis by reducing M2 macrophage infiltration, yet overall it still plays a role in promoting cancer progression.

*FCRL3* exerts regulatory functions in immune responses by recruiting inhibitory molecules through its cytoplasmic domain, thereby reducing the activation capacity of T cells. This immunosuppressive mechanism may influence immune infiltration in gastric cancer, particularly in the MSI and EBV subtypes (Bianchi et al., 2026). EFEMP1 has been identified as a metastatic biomarker in osteosarcoma, promoting lung metastasis and associated with poor prognosis. These mechanisms may be relevant to the metastatic process in gastric cancer (Almeida et al., 2023), especially in the CIN subtype, which is commonly characterized by chromosomal instability and distant metastasis.

In summary, although numerous previous studies have employed WGCNA to identify key genes associated with gastritis or gastric cancer, the present study specifically focuses on the molecular mechanisms underlying the transition from gastritis to early intestinal-type gastric cancer. By integrating multiple datasets, we systematically screened potential key genes involved in this multistep process, providing novel insights into the stage-specific molecular dynamics of gastric carcinogenesis and highlighting candidate targets for intercepting precancerous progression and developing therapeutic interventions. However, it should be noted that our findings are primarily relevant to the intestinal subtype of gastric cancer, which follows a distinct pathogenetic pathway (e.g., the Correa cascade) compared to diffuse or other subtypes. Therefore, the identified mechanisms and gene signatures may not be generalizable to other histological or molecular subtypes of gastric cancer. Furthermore, this study relies entirely on publicly available datasets and computational validation. The lack of experimental or clinical validation represents a significant limitation, which may affect the biological significance and translational potential of our findings.

## Data Availability

Publicly available datasets were analyzed in this study. This data can be found in the Gene Expression Omnibus (GEO) repository (http://www.ncbi.nlm.nih.gov/geo/), with accession number GSE130823 (https://www.ncbi.nlm.nih.gov/geo/query/acc.cgi?acc=GSE130823) and GSE55696 (https://www.ncbi.nlm.nih.gov/geo/query/acc.cgi?acc=GSE55696).
